# Supplementary material for: Biologically Relevant Murine Models of Chronic Pseudomonas aeruginosa Respiratory Infection
Source: Pathogens. 2023 Aug 17;12(8):1053. doi: 10.3390/pathogens12081053 (PMC10458525; doi:10.3390/pathogens12081053)
Supplement: Supplementary file 1 [file pathogens-12-01053-s001.zip › pathogens-2459411-supplementary.pdf]

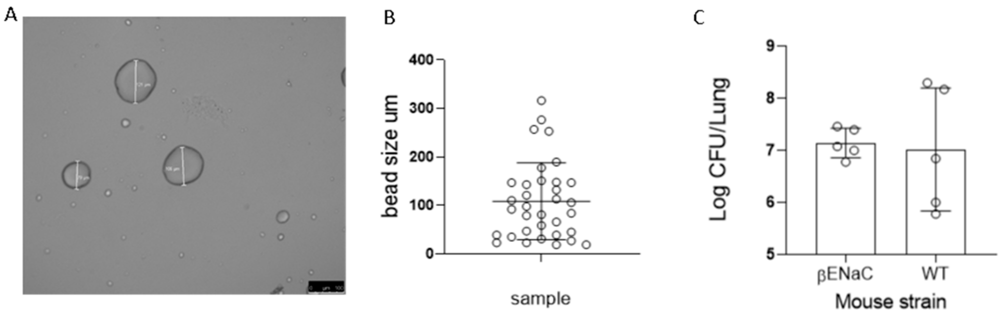

Figure S1. Despite utilising published protocols and a variety of optimisation steps, we found that prepared agar beads (**A**) showed significant diversity in size (**B**). Whilst the inoculation of βENaC mice resulted in consistent establishment of chronic infection (7 days post-inoculation), there was an unacceptable degree of variability in wild-type (C57BL6) animals (**C**) .O represent individual data points

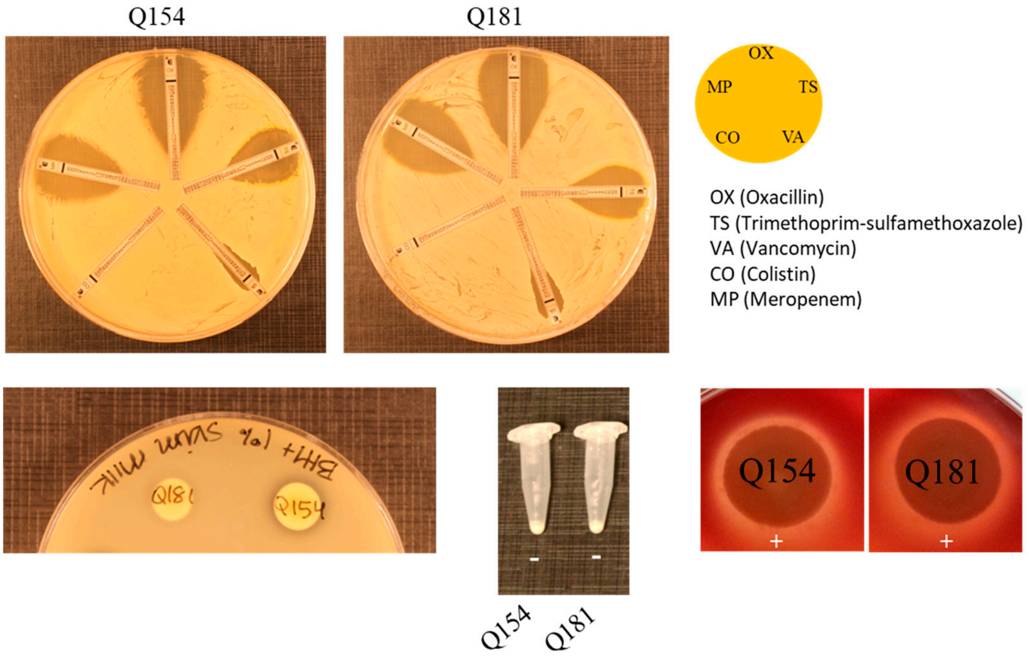

Figure S2. Phenotypic characterisation of *S. aureus* isolates.

Table S1; Characterisation of divergent clinical isolates of *S. aureus.*

Strain	Protease	Hemolytic (sheep blood)	Carotenoid pigment production	mecA gene	Antibiotic sensitivity				
					Oxacillin	Vancomycin	Colistin	Meropenem	Trimethoprim-sulfamethoxazole
Q154	+	+	-	-	0.23	7.3	>256	0.1	0.64
Q181	-	+	-	-	0.25	6	>256	0.06	0.29

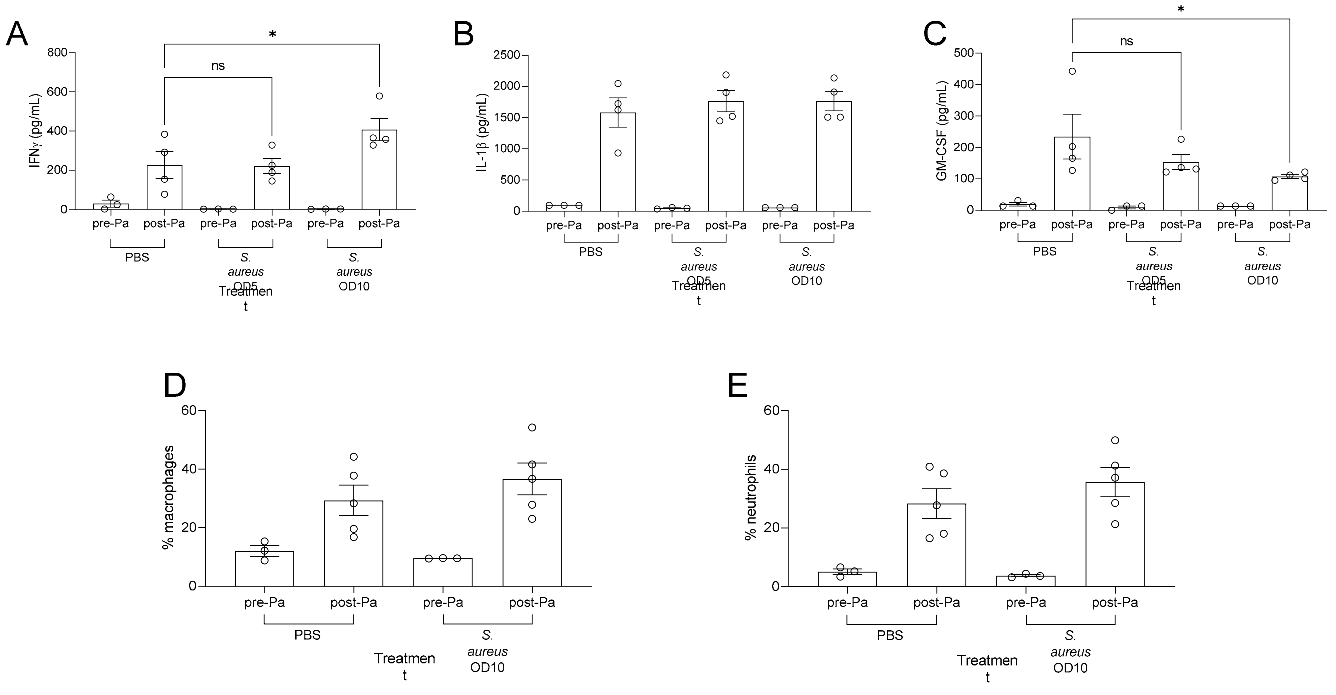

Figure S3. There was Increased levels of IFNγ (A) with *S. aureus* pre-treatment, reduced G-CSF (C) and no difference in IL-1β (B) – despite changes in cytokines and CFU, there is no significant difference in macrophages or neutrophils. O represent individual data points, * $p \leq 0.05$, ** $p \leq 0.01$, *** $p \leq 0.001$, **** $p \leq 0.0001$.

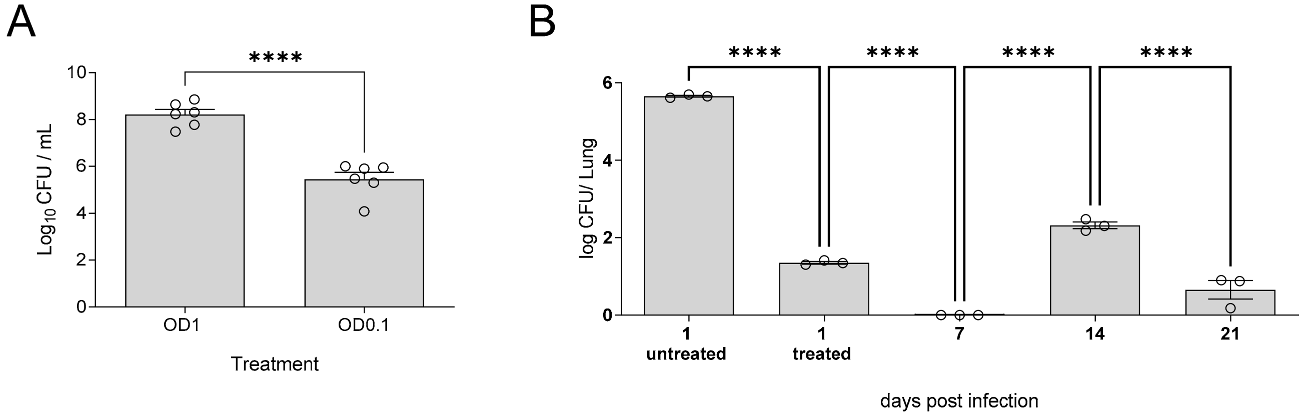

Figure S4. (**A**) Mice were infected intranasally with high (OD1 = ~1x10^6) or low (OD0.1 = ~1x10^5) dose PAO1; significantly less CFU were detected in lungs of the mice infected with the low dose. (**B**) After low dose infection, followed by 5 days of antibiotic treatment, complete clearance was achieved at days 7, whilst there was bounce back at day 14; however, the level was not sustained at day 21. O represent individual data points, **** $p \leq 0.0001$.
